# Background risk of breast cancer and the association between physical activity and mammographic density

**DOI:** 10.1186/s13058-015-0565-4

**Published:** 2015-04-02

**Authors:** Thang Trinh, Mikael Eriksson, Hatef Darabi, Stephanie E Bonn, Judith S Brand, Jack Cuzick, Kamila Czene, Arvid Sjölander, Katarina Bälter, Per Hall

**Affiliations:** Department of Medical Epidemiology and Biostatistics, Karolinska Institutet, Box 281, Stockholm, 17177 Sweden; Centre for Cancer Prevention, Wolfson Institute of Preventive Medicine, Queen Mary University of London, Charterhouse Square, London, EC1M 6BQ UK

## Abstract

**Introduction:**

High physical activity has been shown to decrease the risk of breast cancer, potentially by a mechanism that also reduces mammographic density. We tested the hypothesis that the risk of developing breast cancer in the next 10 years according to the Tyrer-Cuzick prediction model influences the association between physical activity and mammographic density.

**Methods:**

We conducted a population-based cross-sectional study of 38,913 Swedish women aged 40–74 years. Physical activity was assessed using the validated web-questionnaire Active-Q and mammographic density was measured by the fully automated volumetric Volpara method. The 10-year risk of breast cancer was estimated using the Tyrer-Cuzick (TC) prediction model. Linear regression analyses were performed to assess the association between physical activity and volumetric mammographic density and the potential interaction with the TC breast cancer risk.

**Results:**

Overall, high physical activity was associated with lower absolute dense volume. As compared to women with the lowest total activity level (<40 metabolic equivalent hours [MET-h] per day), women with the highest total activity level (≥50 MET-h/day) had an estimated 3.4 cm^3^ (95% confidence interval, 2.3-4.7) lower absolute dense volume. The inverse association was seen for any type of physical activity among women with <3.0% TC 10-year risk, but only for total and vigorous activities among women with 3.0-4.9% TC risk, and only for vigorous activity among women with ≥5.0% TC risk. The association between total activity and absolute dense volume was modified by the TC breast cancer risk (*P*_interaction_ = 0.05). As anticipated, high physical activity was also associated with lower non-dense volume. No consistent association was found between physical activity and percent dense volume.

**Conclusions:**

Our results suggest that physical activity may decrease breast cancer risk through reducing mammographic density, and that the physical activity needed to reduce mammographic density may depend on background risk of breast cancer.

## Introduction

Primary prevention of breast cancer is a major challenge since most of the established risk factors, such as family history, age at menarche and number of childbirths, are difficult to influence. However, physical activity is a modifiable lifestyle factor that has consistently been shown to reduce breast cancer risk [1-4]. According to a recent meta-analysis, the most physically active women had a significant 12% lower breast cancer risk compared with the least active women [5].

Mammographic density is one of the strongest risk factors of breast cancer [6,7]. Women with high mammographic density have a fourfold to sixfold increased risk compared with women with low density [6,8]. Some studies have shown that women who are more physically active have a lower mammographic density compared with less active women [9-11], whereas others have found no association [12-19]. Reasons for null findings could be small sample sizes or differences in characteristics of study populations.

Moreover, no study has assessed whether the background risk of breast cancer modifies the association between physical activity and mammographic density. The Tyrer–Cuzick (TC) prediction model estimates individual risk of developing breast cancer within the following 10 years based on several established risk factors of breast cancer [20]. Mammographic density is currently not included in the TC model.

We examined the association between physical activity and volumetric mammographic density and the potential effect measure modification by the TC 10-year breast cancer risk in 38,913 Swedish women.

## Methods

### Study population

The KARolinska MAmmography Project for Risk Prediction of Breast Cancer (KARMA) is a population-based prospective cohort study of women attending one of four mammography units in the national mammography screening program in Sweden [21]. The participants responded to a detailed web-based questionnaire including information on breast cancer risk factors. Raw and processed full-field digital mammograms have been stored.

Women aged 40 to 74 years with baseline mammograms (*n* = 50,599) were included. Women were excluded if they had incomplete questionnaire answers (*n* = 5,302), missing information on age (*n* = 144) or body mass index (BMI; *n* = 215), previous cancers other than nonmelanoma skin cancer (*n* = 2,765), breast enlargement, reduction or surgery (*n* = 2,070), recent pregnancy (*n* = 46) or mammogram from only one breast (*n* = 1,144). The final analyses included 38,913 women.

The ethical review board at the Karolinska Institutet approved the study. All participants provided written informed consent.

### Mammographic density measurement

Mammograms from cranial–caudal and mediolateral oblique views were obtained using full-field digital mammography systems. We used raw mammograms from the mediolateral oblique view, which is the routine projection during mammography screening in Sweden. A high correlation has been observed between mammographic density measures using mammograms from cranial–caudal and mediolateral oblique views in a published study using the KARMA dataset [22].

Volumetric mammographic density was measured using the fully automated Volpara method. Technical details of the method have been described elsewhere [23]. Briefly, the algorithm computes the thickness of dense tissue at each pixel using the X-ray attenuation of an entirely fatty region as an internal reference. The absolute dense volume (cm^3^) is calculated by integrating the dense thickness at each pixel over the whole mammogram, and the total breast volume (cm^3^) is obtained by multiplying the breast area by breast thickness, with an appropriate correction at the breast edge. Percent dense volume (%) is calculated as the ratio of these two measures. For analyses, we calculated the average mammographic density of the left and right breasts. Our group has recently shown that Volpara density measures are highly correlated with those obtained using the Cumulus method [22,24] and predict breast cancer risk [22].

### Assessment of physical activity

Physical activity data were collected using the self-administrated questionnaire Active-Q [25]. The participants reported the type, frequency and duration of physical activities performed during the past months prior to questionnaire completion. The questionnaire includes four domains of activity; daily occupation, transportation to and from occupation, leisure-time activity and sports, and sleep duration.

We used the duration and frequency of each activity to calculate the average daily duration in hours (hours/day), which was multiplied by a corresponding metabolic equivalent of task (MET) value [26] to obtain daily MET-hours (MET-hours/day). One MET-hour is equivalent to the energy expenditure of quiet resting during 1 hour. MET-hour values from individual activities, including sleep, were summarized into total activity. If the total duration of all reported activities differed from 24 hours, time was added or subtracted to acquire the total activity during 24 hours. Each hour added or subtracted was assigned a MET of 2.0 [25]. We also assessed the daily duration of moderately intense activity including occupational activity (MET = 3.0 to 6.0), such as walking and leisurely bicycling, and vigorously intense activity (MET >6.0), such as jogging, running and fast bicycling. Recreational activity was defined as the daily duration of activity performed at a moderate to vigorous intensity during leisure time and sports.

### Covariates

The self-reported questionnaire includes extensive information on factors that have been suggested to be associated with physical activity and mammographic density. Factors used for adjustment are described in Statistical analysis. Women reporting menstruations in the 12 months prior to study entry were considered premenopausal. Women reporting no menstruation or oophorectomy were considered postmenopausal. Women with missing menstruation status or having no menstruation due to gynecological surgery other than oophorectomy were considered premenopausal if ≤55 years or postmenopausal if >55 years. In addition, an individual 10-year breast cancer risk was calculated using the TC prediction model [20]. The model incorporates information on family history of breast cancer and personal characteristics including age at menarche, parity and age at first childbirth, age at menopause, proliferative benign breast diseases, atypical hyperplasia, lobular carcinoma *in situ*, height and BMI.

### Statistical analysis

Explanatory variables included total activity as well as moderate, vigorous and moderate–vigorous recreational activity. We considered physical activities both as continuous and categorical exposures, respectively. In the latter, total activity was categorized as <40.0, 40.0 to 44.9, 45.0 to 49.9 and ≥50 MET-hours/day. Moderate activity was categorized as <2.0, 2.0 to 4.9, 5.0 to 6.9 and ≥7.0 hours/day. Vigorous activity was categorized as <0.25, 0.25 to 0.49, 0.5 to 0.9 and ≥1.0 hours/day. Moderate–vigorous recreational activity was categorized as <0.5, 0.5 to 0.9, 1.0 to 2.9 and ≥3.0 hours/day. These cutoff points were chosen so that the lowest category of vigorous and recreational activity corresponds to the minimal recommended amount of physical activity for cancer prevention [27].

Outcomes included absolute dense volume, nondense volume and percent dense volume. Linear regressions were performed to estimate the unadjusted and adjusted regression coefficients with 95% confidence intervals (CIs). The lowest physical activity category was used as the reference. We also calculated *P* values for testing the null hypothesis of no exposure–outcome association, referred to as *P*_trend_ (one degree of freedom) and *P*_global_ (three degrees of freedom) for the continuous and categorized exposure models, respectively. To avoid assuming normally distributed error terms and homoscedastic variance, we utilized robust sandwich standard errors [28] to calculate 95% CIs and *P* values. We used two-sided tests with 5% significance level.

In multivariable-adjusted analyses, we adjusted for several potential confounding factors: age at mammography (5-year categories), BMI (<25.0, 25.0 to 29.9, ≥30.0 kg/m^2^), family history of breast cancer in mother or sisters (yes, no), age at menarche (<13, 13, 14, ≥15 years), parity and age at first birth (nulliparous; one or two births, age at first birth <26 years; one or two births, age at first birth ≥26 years; ≥3 births, age at first birth <26 years; ≥3 births, age at first birth ≥26 years), oral contraceptive use (never, ever), menopausal status, use of hormone replacement therapy (HRT; never, past only, current), education level (secondary school, high school, university or higher, other), smoking status (never, past, current) and alcohol consumption (none, 0.1 to 9.9, 10.0 to 29.9, ≥30.0 g/day).

We examined the potential interaction with TC 10-year breast cancer risk by stratifying on 10-year risk and by adding a product term between physical activity and breast cancer risk to the multivariable-adjusted models. For TC breast cancer risk, we used cutoff points similar to the established cutoff points [29]. However, the cutoff point for the highest risk category was set as ≥5.0% in order to have sufficient numbers to conduct stratified analyses. The TC 10-year breast cancer risk was therefore categorized as <3.0, 3.0 to 4.9 and ≥5.0%.

The analyses were performed using the statistical software R version 3.1.0 (R Core Team (2014), Vienna, Austria).

## Results

The mean age at mammography screening was 55.5 (standard deviation, 9.8) years and the mean BMI was 25.4 (standard deviation, 4.2) kg/m^2^ (Table 1). Approximately 57% of the participants were postmenopausal, of whom 5% were currently using HRT. A majority (80%) of the participants had used oral contraceptives. The mean absolute dense volume was 64.2 cm^3^ (95% CI, 63.9 to 64.5) and the mean percent dense volume was 9.0% (95% CI, 9.0 to 9.1). On average, women with higher total activity were younger, had a lower BMI and more childbirth, were more likely to have used oral contraceptives and be currently using HRT, and less likely to smoke and consume alcohol.

**Table 1:** **Characteristics of the study population by level of total activity**

	**All women**	**Total activity**			
**Characteristic**		**<40.0 MET-hours/day**	**40.0 to 44.9 MET-hours/day**	**45.0 to 49.9 MET-hours/day**	**≥50.0 MET-hours/day**
Number (%) of participants	38,913 (100)	12,180 (31.3)	15,273 (39.2)	7,591 (19.5)	3,869 (9.9)
Age at mammography screening (years), mean (SD)	55.5 (9.8)	56.6 (10.0)	56.4 (10.1)	53.6 (9.1)	51.8 (8.0)
Body mass index (kg/m^2^), mean (SD)	25.4 (4.2)	26.1 (4.5)	25.2 (4.0)	24.9 (4.0)	25.2 (4.2)
Age at menarche (years), mean (SD)^a^	13.1 (1.5)	13.1 (1.5)	13.1 (1.5)	13.1 (1.5)	13.1 (1.5)
Absolute dense volume (cm^3^), mean (95% CI)	64.2 (63.9 to 64.5)	66.1 (65.6 to 66.7)	63.4 (62.9 to 63.9)	63.0 (62.3 to 63.7)	63.8 (62.8 to 64.7)
Nondense volume (cm^3^), mean (95% CI)	793.2 (788.6 to 797.7)	865.3 (856.8 to 873.7)	778.4 (771.4 to 785.4)	726.8 (717.1 to 736.6)	754.6 (740.0 to 769.1)
Total breast volume (cm^3^), mean (95% CI)	857.4 (852.7 to 862.0)	931.3 (922.7 to 940.0)	841.8 (834.6 to 849.0)	789.8 (779.9 to 799.8)	818.3 (803.3 to 833.3)
Percent dense volume (%), mean (95% CI)	9.0 (9.0 to 9.1)	8.5 (8.4 to 8.6)	9.0 (8.9 to 9.1)	9.6 (9.5 to 9.7)	9.5 (9.3 to 9.7)
Nulliparous (%)^a^	12.2	15.2	11.4	9.8	11.1
Parous women only (*n*)	34,093	10,307	13,516	6,837	3,433
Age at first birth (years), mean (SD)^a^	27.0 (5.2)	27.0 (5.2)	27.2 (5.2)	27.2 (5.1)	26.5 (5.0)
Number of childbirth, mean (SD)	2.2 (0.8)	2.1 (0.8)	2.2 (0.8)	2.3 (0.8)	2.3 (0.9)
Family history of breast cancer (%)^a^	13.6	14.2	13.7	12.9	13.0
Education level (%)^a^					
Secondary school	13.7	14.9	14.2	11.6	12.0
High school	30.6	30.4	27.9	29.9	43.2
University or higher	52.5	51.6	54.4	55.9	41.1
Other	2.9	2.8	3.2	2.5	3.3
OC use, ever (%)^a^	80.4	80.9	79.4	81.0	81.4
Premenopausal (%)	43.4	39.4	40.3	49.7	56.2
Postmenopausal women only (*n*)	22,009	7,377	9,121	3,817	1,694
Age at menopause (years), mean (SD)	49.9 (5.3)	49.9 (5.3)	50.0 (5.4)	49.9 (5.1)	49.5 (5.4)
HRT use (%)^a^					
Never	62.2	60.8	60.7	65.3	69.0
Past	22.4	23.3	23.9	19.9	16.5
Current	5.1	5.2	4.8	5.7	5.6
Smoking status (%)^a^					
Never	47.8	45.1	48.5	50.3	48.5
Past	39.9	41.4	40.1	38.1	38.0
Current	12.1	13.3	11.1	11.4	13.3
Alcohol consumption (g/day)^a^					
None (%)	17.9	17.8	16.6	17.7	23.7
0.1 to 9.9 (%)	61.2	58.2	62.4	63.9	60.7
10.0 to 29.9 (%)	16.3	18.1	16.8	14.4	11.8
≥ 30.0 (%)	2.7	3.7	2.4	2.3	1.6

^a^Percentage of women with missing values on age at menarche (1.9%), parity status (0.1%), age at first birth (5.8%), family history of breast cancer (2.7%), education level (0.3%), OC use (0.5%), HRT use (10.2%), smoking status (0.2%) and alcohol intake (1.9%). CI, confidence interval; HRT, hormone replacement therapy; MET, metabolic equivalent of task; OC, oral contraceptives; SD, standard deviation.

Women with ≥3.0% TC 10-year risk of developing breast cancer were older, of higher BMI, younger at menarche and older at first birth, more likely to be nulliparous, to have a family history of breast cancer, to be postmenopausal, to be currently using HRT, to have ever smoked and to consume alcohol as compared with women with <3.0% TC risk (Table 2).

**Table 2:** **Characteristics of the study population according to 10-year breast cancer risk estimated by the Tyrer–Cuzick prediction model**

	**Breast cancer risk**		
**Characteristic**	**<3.0%**	**3.0 to 4.9%**	**≥5.0%**
Number of participants (%)	19,633 (50.5)	13,785 (35.4)	5,495 (14.1)
Age at mammography screening (years), mean (SD)	52.8 (10.4)	57.9 (8.5)	58.8 (7.8)
Body mass index (kg/m^2^), mean (SD)	25.1 (4.2)	25.7 (4.2)	25.8 (4.1)
Age at menarche (years), mean (SD)	13.2 (1.5)	13.0 (1.4)	12.9 (1.4)
Nulliparous (%)	8.4	16.0	16.5
Age at first birth (years), mean (SD)^a^	26.0 (5.0)	28.2 (5.0)	28.4 (5.2)
Number of childbirths, mean (SD)^a^	2.3 (0.8)	2.1 (0.8)	2.1 (0.8)
Family history of breast cancer (%)	1.2	9.9	67.2
Education level (%)			
Secondary school	14.1	13.6	12.6
High school	33.9	27.5	26.7
University or higher	49.0	55.5	57.5
Other	2.7	3.2	3.1
OC use, ever (%)	80.9	79.9	79.8
Premenopausal (%)	56.7	30.8	27.9
Age at menopause (years), mean (SD)^b^	49.5 (5.5)	50.2 (5.2)	50.3
HRT use (%)^b^			
Never	63.1	61.4	62.2
Past	22.4	22.2	22.8
Current	3.8	6.0	5.9
Smoking status (%)			
Never	49.9	46.2	44.3
Past	36.9	42.7	43.7
Current	12.9	11.0	11.9
Alcohol consumption (grams/day)			
None (%)	19.3	16.4	16.3
0.1 to 9.9 (%)	61.6	61.3	59.5
10.0 to 29.9 (%)	14.7	17.5	18.8
≥ 30.0 (%)	2.1	3.3	3.5

HRT, hormone replacement therapy; OC, oral contraceptives; SD, standard deviation. ^a^Among parous women only. ^b^Among postmenopausal women only.

The mean total activity level was 43.0 (standard deviation 5.7) MET-hours/day. The participants reported spending on average 2.2, 0.2 and 2.3 hours/day in moderate, vigorous and recreational activity, respectively (data not shown).

In multivariable-adjusted analyses, higher levels of all types of physical activity were associated with lower absolute dense volume (Table 3). The association was most pronounced for total and vigorous activities. Women with the highest total activity level (≥50 MET-hours/day) had an estimated 3.4 cm^3^ (95% CI, 2.3 to 4.7) lower absolute dense volume compared with women with the lowest total activity (<40 MET-hours/day). Women spending ≥1.0 hour/day in vigorous activity had an estimated 3.1 cm^3^ (95% CI, 1.0 to 5.3) lower absolute dense volume compared with those reporting <0.25 hours/day. When physical activity was used as a continuous exposure, each additional 5 MET-hours/day in total activity was associated with a decrease of 1.1 cm^3^ (95% CI, 0.7 to 1.3) in absolute dense volume; and each additional 1 hour/day in vigorous activity was associated with a reduction of 3.9 cm^3^ (95% CI, 2.9 to 4.9) in absolute dense volume.

**Table 3:** **Associations between physical activity and volumetric mammographic density among all women**

**Type of activity**	***N***	**%**	**Absolute dense volume (cm** ^**3**^ **)**		**Nondense volume (cm** ^**3**^ **)**		**Percent dense volume (%)**	
			**Unadjusted**	**Adjusted**	**Unadjusted**	**Adjusted**	**Unadjusted**	**Adjusted**
			**β (95% CI)**	**β (95% CI)** ^**a**^	**β (95% CI)**	**β (95% CI)** ^**a**^	**β (95% CI)**	**β (95% CI)** ^**a**^
Total activity (MET-hours/day)								
< 40.0	12,180	31.3	Reference	Reference	Reference	Reference	Reference	Reference
40.0 to 44.9	15,273	39.2	−2.7 (−3.5, −2.0)	−1.2 (−2.0, −0.4)	−86.9 (−97.8, −75.9)	−14.6 (−23.2, −6.0)	0.5 (0.4, 0.6)	0 (−0.1, 0.1)
45.0 to 49.9	7,591	19.2	−3.1 (−4.0, −2.2)	−2.5 (−3.5, −1.5)	−138.4 (−151.3, −125.5)	−34.9 (−45.0, −24.9)	1.1 (0.9, 1.2)	0.1 (0, 0.2)
≥ 50.0	3,869	9.9	−2.4 (−3.5, −1.3)	−3.4 (−4.7, −2.3)	−110.7 (−127.6, −93.8)	−23.7 (−36.9, −10.4)	1.0 (0.8, 1.2)	0 (−0.2, 0.2)
*P*_global_^b^			<0.001	<0.001	<0.001	<0.001	<0.001	0.39
For every 5 MET-hours/day increase^c^			−0.9 (−1.2, −0.6)	−1.1 (−1.3, −0.7)	−39.0 (−43.1, −35.0)	−8.5 (−12.0, −5.5)	0.3 (0.2, 0.4)	0 (−0.04, 0.04)
*P*_trend_^d^			<0.001	<0.001	<0.001	<0.001	<0.001	0.96
Moderate activity (hours/day)								
< 2.0	15,104	38.8	Reference	Reference	Reference	Reference	Reference	Reference
2.0 to 4.9	12,300	31.6	−2.4 (−3.2, −1.7)	−0.3 (−1.1, 0.5)	−43.3 (−54.1, −32.5)	−4.8 (−13.3, 3.6)	0.1 (0, 0.2)	0 (−0.1, 0.1)
5.0 to 6.9	5,729	14.7	−1.1 (−2.0, −0.1)	−1.0 (−2.1, 0)	−27.6 (−41.5, −13.8)	3.1 (−7.8, 14.0)	0.1 (0. 0.3)	−0.2 (−0.3, 0)
≥ 7.0	5,780	14.9	−1.0 (−1.9, 0)	−1.9 (−2.9, −0.9)	−63.1 (−77.0, −49.2)	−5.1 (−15.9, 5.7)	0.7 (0.5, 0.8)	−0.1 (−0.2, 0.1)
*P*_global_^b^			<0.001	<0.001	<0.001	0.41	<0.001	0.03
For every 1 h/day increase^c^			−0.1 (−0.2, 0.02)	−0.3 (−0.4, −0.1)	−7.9 (−9.6, −6.2)	−0.5 (−1.8, 0.8)	0.1 (0.07, 0.1)	−0.02 (−0.03, 0)
*P*_trend_^d^			0.12	<0.001	<0.001	0.44	<0.001	0.09
Vigorous activity (hours/day)								
< 0.25	28,559	73.4	Reference	Reference	Reference	Reference	Reference	Reference
0.25 to 0.49	6,237	16.0	−2.6 (−3.5, −1.7)	−2.6 (−3.5, −1.6)	−145.1 (−156.6, −133.6)	−43.1 (−52.1, −34.1)	1.2 (1.1, 1.4)	0.2 (0.1, 0.4)
0.5 to 0.9	3,226	8.3	−4.9 (−6.0, −3.8)	−3.5 (−4.7, −2.3)	−163.2 (−178.1, −148.3)	−64.5 (−76.5, −52.5)	1.2 (1.0, 1.3)	0.4 (0.2, 0.6)
≥ 1.0	891	2.3	−5.3 (−7.4, −3.3)	−3.1 (−5.3, −1.0)	−134.9 (−164.0, −105.9)	−60.6 (−82.9, −38.4)	0.9 (0.6, 1.3)	0.6 (0.3, 0.9)
*P*_global_^b^			<0.001	<0.001	<0.001	<0.001	<0.001	<0.001
For every 1 h/day increase^c^			−5.6 (−6.5, −4.6)	−3.9 (−4.9, −2.9)	−181.4 (−199.9, −162.8)	−62.7 (−75.6, −49.9)	1.3 (1.1, 1.5)	0.4 (0.2, 0.6)
*P*_trend_^d^			<0.001	<0.001	<0.001	<0.001	<0.001	<0.001
Recreational activity (hours/day)								
< 0.5	2,282	5.9	Reference	Reference	Reference	Reference	Reference	Reference
0.5 to 0.9	5,170	13.3	−0.5 (−2.1, 1.0)	−0.7 (−2.3, 1.0)	−91.2 (−115.3, −67.0)	−40.4 (−59.7, −21.2)	0.7 (0.4, 0.9)	0.2 (0, 0.4)
1.0 to 2.9	20,860	53.6	−2.5 (−3.9, −1.2)	−1.6 (−3.0, −0.1)	−138.0 (−159.5, −116.6)	−49.4 (−66.7, −32.2)	1.0 (0.8, 1.2)	0.3 (0.1, 0.5)
≥ 3.0	10,601	27.2	−4.3 (−5.7, −2.9)	−2.5 (−4.0, −1.0)	−160.7 (−183.0, −138.4)	−50.5 (−68.6, −32.5)	1.1 (0.9, 1.3)	0.3 (0.1, 0.5)
*P*_global_^b^			<0.001	<0.001	<0.001	<0.001	<0.001	0.02
For every 1 hour/day increase^c^			−0.9 (−1.1, −0.7)	−0.5 (−0.7, −0.3)	−19.3 (−22.1, −16.6)	−4.4 (−6.7, −2.1)	0.1 (0.08, 0.1)	0.02 (0, 0.05)
*P*_trend_^d^			<0.001	<0.001	<0.001	<0.001	<0.001	0.09

β, regression coefficient; CI, confidence interval; MET, metabolic equivalent of task. ^a^Adjusted for age at mammography (5-year categories), body mass index (<25.0, 25.0 to 29.9, ≥30.0 kg/m^2^), family history of breast cancer in mother or sisters (yes, no), age at menarche (<13, 13, 14, ≥15 years), parity and age at first birth (nulliparous; one or two births, age at first birth <26 years; one or two births, age at first birth ≥26 years; ≥3 births, age at first birth <26 years; ≥3 births, age at first birth ≥26 years), oral contraceptives use (never, ever), menopausal status (premenopausal, postmenopausal), use of hormone replacement therapy (never, past, current), education level (secondary school, high school, university or higher, other), smoking status (never, past, current) and alcohol consumption (none, 0.1 to 9.9, 10.0 to 29.9, ≥30.0 g/day). ^b^ *P*_global_ values were obtained from regression models using physical activity as a categorical exposure. ^c^Change in volumetric mammographic density for every 5 MET-hours/day increase in total activity or every 1 hour/day increase in moderate, vigorous or recreational activity, respectively; from regression models using physical activity as a continuous exposure. ^d^ *P*_trend_ values were obtained from regression models using physical activity as a continuous exposure.

Total, vigorous and recreational activities were also associated with lower nondense volume (Table 3). Women devoting ≥0.25 hours/day to vigorous activity had 60.6 cm^3^ (95% CI, 38.4 to 82.9) lower nondense volume compared with those reporting <0.25 hours/day of vigorous activity. In contrast, vigorous and recreational activities were associated with higher percent dense volume.

In models stratified by the TC 10-year breast cancer risk (Table 4), an inverse association with absolute dense volume was seen for any type of activity among women with <3.0% TC risk, but only for total and vigorous activities among women with 3.0 to 4.9% TC risk, and only for vigorous activity among women with ≥5.0% TC risk. The formal test for interaction with breast cancer risk was statistically significant for total activity (*P*_interaction_ = 0.05). Among women with <3.0% TC risk, a decrease in absolute dense volume was found for women with any higher levels of total activity compared with the lowest level (<40.0 MET-hours/day). However, a decrease in absolute dense volume was only seen among women with 3.0 to 4.9% TC risk performing 45.0 to 49.9 or ≥50.0 MET-hours/day and only in women with ≥5.0% TC risk engaging in ≥50.0 MET-hours/day, as compared with the lowest category.

**Table 4:** **Multivariable associations between physical activity and absolute dense volume (cm** ^**3**^ **) stratified by 10-year breast cancer risk estimated by the Tyrer–Cuzick prediction model**

	**Breast cancer risk <3.0%**			**Breast cancer risk 3.0 to 4.9%**			**Breast cancer risk ≥5.0%**		
**Type of activity**	***N***	**%**	**Multivariable-adjusted**	***N***	**%**	**Multivariable-adjusted**	***N***	**%**	**Multivariable-adjusted**
			**β (95% CI)** ^**a**^			**β (95% CI)** ^**a**^			**β (95% CI)** ^**a**^
Total activity (MET-hours/day)									
< 40.0	5,791	29.5	Reference	4,499	32.6	Reference	1,890	34.4	Reference
40 to 44.9	7,515	38.3	−1.9 (−3.1, −0.7)	5,567	40.4	−0.3 (−1.7, 1.0)	2,191	39.9	−0.7 (−2.7, 1.3)
45.0 to 49.9	4,072	20.7	−3.3 (−4.7, −1.9)	2,520	18.3	−2.0 (−3.6, −0.4)	999	18.2	−0.5 (−3.2, 2.1)
≥ 50.0	2,255	11.5	−4.2 (−5.8, −2.6)	1,199	8.7	−2.2 (−4.4, 0)	415	7.6	−3.7 (−7.1, −0.3)
*P*_global_^b^			<0.001			0.04			0.2
For every 5 MET-hours/day increase^c^			−1.2 (−1.5, −0.8)			−0.7 (−1.2, −0.2)			−1.0 (−1.9, −0.3)
*P*_trend_^d^			<0.001			0.01			0.01
*P*_interaction_^e^			0.05						
Moderate activity (hours/day)									
< 2.0	7,173	36.5	Reference	5,623	40.8	Reference	2,308	42.0	Reference
2.0 to 4.9	6,100	31.1	0 (−1.2, 1.2)	4,431	32.1	−0.4 (−1.7, 0.9)	1,769	32.2	−0.7 (−2.7, 1.3)
5.0 to 6.9	3,061	15.6	−1.1 (−2.6, 0.3)	1,920	13.9	−0.7 (−2.4, 1.0)	748	13.6	−0.7 (−3.5, 2.1)
≥ 7.0	3,299	16.8	−2.4 (−3.8, −1.0)	1,811	13.1	−1.1 (−2.9, 0.7)	670	12.2	−0.8 (−3.7, 2.0)
*P*_global_			<0.001			0.65			0.88
For every 1 hour/day increase^c^			−0.3 (−0.5, −0.1)			−0.1 (−0.4, 0.1)			−0.1 (−0.5, 0.2)
*P*_trend_^d^			<0.001			0.14			0.40
*P*_interaction_^e^			0.13						
Vigorous activity (hours/day)									
< 0.25	14,190	72.3	Reference	10,262	74.4	Reference	4,107	74.7	Reference
0.25 to 0.49	3,293	16.8	−2.8 (−4.1, −1.5)	2,118	15.4	−2.3 (−3.9, −0.7)	826	15.0	−2.2 (−4.7, 0.4)
0.5 to 0.9	1,699	8.7	−3.3 (−5.0, −1.7)	1,084	7.9	−3.3 (−5.3, −1.2)	443	8.1	−4.4 (−7.2, −1.6)
≥ 1.0	451	2.3	−6.3 (−9.1, −3.5)	321	2.3	0.5 (−3.0, 4.1)	119	2.2	−0.2 (−5.9, 5.5)
*P*_global_			<0.001			<0.001			0.01
For every 1 hour/day increase^c^			−5.5 (−7.0, −4.1)			−1.7 (−3.3, −0.03)			−3.7 (−6.2, −1.1)
*P*_trend_^d^			<0.001			0.05			0.01
*P*_interaction_^e^			0.11						
Recreational activity (hours/day)									
< 0.5	1,068	5.4	Reference	866	6.3	Reference	348	6.3	Reference
0.5 to 0.9	2,450	12.5	0.8 (−1.7, 3.2)	1,906	13.8	−1.4 (−4.2, 1.3)	814	14.8	−3.0 (−7.0, 1.1)
1.0 to 2.9	10,336	52.6	−1.3 (−3.4, 0.7)	7,514	54.5	−1.8 (−4.2, 0.6)	3,010	54.8	−1.6 (−5.2, 2.1)
≥ 3.0	5,779	29.4	−2.1 (−4.3, 0.1)	3,499	25.4	−2.4 (−5.0, 0.1)	1,323	24.1	−3.2 (−7.1, 0.7)
*P*_global_			0.01			0.3			0.24
For every 1 hour/day increase^c^			−0.5 (−0.8, −0.2)			−0.3 (−0.6, 0.1)			−0.6 (−1.2, −0.1)
*P*_trend_^d^			<0.001			0.15			0.03
*P*_interaction_^e^			0.37						

β, regression coefficient; CI, confidence interval; MET, metabolic equivalent of task. ^a^Adjusted for age at mammography (5-year categories), body mass index (<25.0, 25.0 to 29.9, ≥30.0 kg/m^2^), family history of breast cancer in mother or sisters (yes, no), age at menarche (<13, 13, 14, ≥15 years), parity and age at first birth (nulliparous; one or two births, age at first birth <26 years; one or two births, age at first birth ≥26 years; ≥3 births, age at first birth <26 years; ≥3 births, age at first birth ≥26 years), oral contraceptives use (never, ever), menopausal status (premenopausal, postmenopausal), use of hormone replacement therapy (never, past, current), education level (secondary school, high school, university or higher, other), smoking status (never, past, current) and alcohol consumption (none, 0.1 to 9.9, 10.0 to 29.9, ≥30.0 g/day). ^b^ *P*_global_ values were obtained from regression models using physical activity as a categorical exposure. ^c^Change in volumetric mammographic density for every 5 MET-hours/day increase in total activity or every 1 hour/day increase in moderate, vigorous or recreational activity, respectively; from regression models using physical activity as a continuous exposure. ^d^ *P*_trend_ values were obtained from regressions models using physical activity as a continuous exposure. ^e^ *P*_interaction_ values were obtained from the nonstratified regression models by adding a product term between physical activity and the 10-year breast cancer risk as predicted using the Tyrer–Cuzick prediction model.

For nondense volume, an inverse association was observed for total and vigorous activities across all categories of breast cancer risk (data not shown). No consistent association was found between physical activity and percent dense volume (data not shown).

## Discussion

Our main finding was that higher levels of physical activity were associated with lower absolute dense volume and nondense volume, but seemed to be associated with higher percent dense volume. A potentially novel finding was that the association between physical activity and absolute dense volume seemed to vary according to the risk of developing breast cancer in the next 10 years as estimated using the TC prediction model. An inverse association with absolute dense volume was seen for any type of activity among women with <3.0% TC 10-year risk of breast cancer, but only for total and vigorous activities among women with 3.0 to 4.9% TC risk and only for vigorous activity among women with ≥5.0% TC risk. The formal test for interaction with TC 10-year breast cancer risk was statistically significant for total activity.

Epidemiological investigations into physical activity and mammographic density have produced inconsistent findings. Direct comparisons between studies are challenging due to differences in methods for measuring mammographic density and the type of physical activity assessed. Percent mammographic density has been reported as the primary density measurement in previous studies, most of which found no association [12-17]. Physical activity was not associated with percent dense area in a study of 1,900 premenopausal and postmenopausal women [19]. Despite the relatively large sample size, this study is limited by the assessment of physical activity based on only one question and the visual estimation of mammographic density. Another study on 1,147 postmenopausal women found no significant differences in either absolute or percent dense areas between women performing recreational physical activity for >2 hours/week versus none [30]. Although this study measured breast density on a continuous scale using the computer-assisted Cumulus method [24], the range of physical activity was narrow. Similarly, a sizable study on 1,394 postmenopausal cancer-free women found no significant difference (odds ratio, 078; 95% CI, 0.45 to 1.34) between the most active and the least active women regarding the likelihood of having high-risk (>50%) percent dense area [13]. However, in this study the physical activity levels were of limited contrast because the activity level was categorized based only on the intensity of occupational activity and the duration of physical activity performed during leisure time (none, <0.5, 0.5 to 1.0, >1 hour/day) [13]. An investigation of 620 women showed a nonsignificant trend of increasing physical activity with higher percent dense area [16]. Of note is that there was also a nonsignificant association between physical activity and lower absolute dense area [16]. Percent breast density is determined by the relative amount of breast dense and nondense (that is, fatty) tissues. A reduction in both dense and nondense tissues, providing the decrease in nondense tissue is more pronounced than in dense tissue, still leads to increased percent density. We thus do not believe that percent density is the optimal measure for the effect of physical activity on mammographic density since high physical activity was associated with lower amounts of both absolute dense volume and nondense volume (Table 3), leading to a higher percent dense volume among more physically active women. Increasing level and increasing duration of physical activity have been shown to decrease body weight and fat mass [31,32]. Because breast fat is highly associated with body fat, the decrease in nondense volume and the increase in percent dense volume observed in our study are probably due to the effect of physical activity on body fat.

In agreement with some studies [9-11], we found high physical activity to be associated with lower absolute amount of breast dense tissue. Absolute mammographic density reflects the amount of fibroglandular tissue in the breast, and thus the number of cells from which breast cancer arises. Studies have shown that not only percent mammographic density but also absolute mammographic density is strongly associated with risk of breast cancer [33,34].

In the only randomized controlled trial to date examining the influences of physical activity on mammographic density, no statistically significant difference was found for changes in the area or volume of absolute and percent dense breast tissues between the controls and the exercisers (aerobic exercise, 45 minutes/time × 5 times/week for 1 year) [17]. It is important to note that that this study includes postmenopausal women who were sedentary at baseline, and thus a higher level of activity may be required to affect mammographic density.

In addition, several studies only focused on specific physical activity – for example, recreational activity [14,15] – and could not thoroughly distinguish between different activity intensities [13-15,30]. Furthermore, physical activity levels have been low in some studies [13,30], and therefore the effect of overall physical activity and high-intensity activity on mammographic density could have been missed. The broad range of activity level in our study allows us to compare the density between women with different activity levels. We showed that not only vigorous activity, but also total activity, was associated with lower mammographic density. The activity level in our study is consistent with the level of self-reported physical activity in a large population-based study of middle-aged Swedish women [35].

To our knowledge, this is the largest study so far investigating the association between a wide range of physical activity and mammographic density in women, and also the first to use volumetric density as the outcome. The majority of previous studies into mammographic density was assessed using subjective (that is, manual) and area-based methods [12-16], in which mammogram pixels represent either complete dense or nondense tissue. We used a fully automated technique to estimate volumetric density, which takes breast thickness into account by measuring the X-ray attenuation arising from different degrees of density in each pixel. Volumetric measures are therefore expected to capture the actual amount of fibroglandular tissue in the breast more precisely, and are theoretically expected to be a better predictor of breast cancer risk than area-based density measures. Indeed, volumetric measures of mammographic density have been shown to predict breast cancer risk more accurately compared with area-bases measures [36].

This study has some limitations. The cross-sectional nature of the study does not permit us to rule out reverse causation between physical activity and mammographic density. It is, however, unlikely that having a high mammographic density could prevent women from being physically active. Self-reported physical activity is prone to misclassification, but it is most probably nondifferential since women were not aware of their mammographic density and the potential association between physical activity and mammographic density.

Physical activity has been hypothesized to reduce breast cancer risk through several hormone-related mechanisms, and could therefore possibly decrease mammographic density. Estrogens are considered to have a major role in stimulating breast epithelial cell proliferation [37]. During the premenopausal period, physical activity could reduce sex hormone levels through delayed menarche and alterations in menstrual function [38]. Physical activity has also been shown to decrease estrogen levels among postmenopausal women, and part of this effect is attributable to reduced body fat [39,40]. After menopause, when the ovaries no longer produce estrogens, endogenous estrogens are primarily produced from the conversion of androgens by aromatase enzyme in adipose tissue [41]. Higher estrogen levels have been linked to greater mammographic density in premenopausal [42] and postmenopausal women [43,44].

To our knowledge, this is the first study to investigate the influence of breast cancer risk on the association between physical activity and mammographic density. The TC prediction model incorporates hormonal factors associated with breast cancer risk, such as parity, age at menarche, first birth and menopause, which also influence a woman’s cumulative sex hormone exposure [45,46]. The risk of breast cancer as estimated using the TC prediction model may thus potentially reflect sex hormone exposure. Studies have shown that women with higher steroid hormone concentrations have an increased risk of breast cancer [47,48]. Physical activity has been shown to affect menstrual cycle characteristics [49,50] and to be inversely associated with estrone and estradiol levels [40,51,52]. More intense physical activity could perhaps be required to influence the higher hormone levels seen in high-risk women. Indeed, it has been reported that intense exercise causes reproductive disruptions in women, including delayed menarche and amenorrhea [53]. Such a mechanism could potentially explain why any type of physical activity was associated with lower absolute mammographic density among women with <3.0% TC 10-year risk, and that the inverse association was only found for vigorous activity among women with ≥5.0% TC 10-year risk.

Primary prevention of breast cancer is of great importance given the increase in breast cancer incidence worldwide [54]. The primary preventive measures currently available range from increased physical activity to prophylactic mastectomy. As a modifiable factor, increasing physical activity could be a feasible approach to reduce breast cancer risk. An association has been noted between high volumetric mammographic density, as measured using the Volpara method, and an increased breast cancer risk among KARMA participants [22]. Based on these findings, the women in our study who spent ≥1.0 hour/day in vigorous activity had an estimated decrease of approximately 2.5% in relative breast cancer incidence rate compared with women spending <0.25 hours/day in vigorous activity.

To be involved in a daily 15 minutes of vigorous physical activity is in line with the latest American Cancer Society Guidelines [27] and the Nordic Nutrition Recommendations [55] indicating that adults should perform at least 150 minutes of moderate intensity or 75 minutes of vigorous intensity physical activity each week to achieve health benefits. We show that women who engaged in vigorous activity, such as jogging, running or fast bicycling, for ≥0.25 hours/day had significantly lower density compared with women reporting <0.25 hours/day, regardless of background breast cancer risk.

## Conclusions

Our findings suggest that the beneficial effect of physical activity on breast cancer risk could be mediated through reducing mammographic density. Our results also suggest that women at high risk of breast cancer may have to engage in more intense physical activity to achieve a density reduction compared with women at lower risk. This is the first study to take background breast cancer risk into consideration when examining the association between physical activity and mammographic density. Future research is needed to confirm our findings.

## Abbreviations

BMI: body mass index
CI: confidence interval
HRT: hormone replacement therapy
KARMA: KARolinska MAmmography Project for Risk Prediction of Breast Cancer
MET: metabolic equivalent of task

## References

[1] Thune I, Brenn T, Lund E, Gaard M (1997). Physical activity and the risk of breast cancer. N Engl J Med..

[2] Bernstein L, Henderson BE, Hanisch R, Sullivan-Halley J, Ross RK (1994). Physical exercise and reduced risk of breast cancer in young women. J Natl Cancer Inst..

[3] McTiernan A, Kooperberg C, White E, Wilcox S, Coates R, Adams-Campbell LL (2003). Recreational physical activity and the risk of breast cancer in postmenopausal women: the Women's Health Initiative Cohort Study. JAMA..

[4] Maruti SS, Willett WC, Feskanich D, Rosner B, Colditz GA (2008). A prospective study of age-specific physical activity and premenopausal breast cancer. J Natl Cancer Inst..

[5] Wu Y, Zhang D, Kang S (2013). Physical activity and risk of breast cancer: a meta-analysis of prospective studies. Breast Cancer Res Treat..

[6] Boyd NF, Lockwood GA, Martin LJ, Knight JA, Byng JW, Yaffe MJ (1998). Mammographic densities and breast cancer risk. Breast Dis..

[7] Boyd NF, Byng JW, Jong RA, Fishell EK, Little LE, Miller AB (1995). Quantitative classification of mammographic densities and breast cancer risk: results from the Canadian National Breast Screening Study. J Natl Cancer Inst..

[8] McCormack VA, dos Santos SI (2006). Breast density and parenchymal patterns as markers of breast cancer risk: a meta-analysis. Cancer Epidemiol Biomarkers Prev..

[9] Irwin ML, Aiello EJ, McTiernan A, Bernstein L, Gilliland FD, Baumgartner RN (2007). Physical activity, body mass index, and mammographic density in postmenopausal breast cancer survivors. J Clin Oncol..

[10] Irwin ML, Aiello EJ, McTiernan A, Baumgartner RN, Baumgartner KB, Bernstein L (2006). Pre-diagnosis physical activity and mammographic density in breast cancer survivors. Breast Cancer Res Treat..

[11] Qureshi SA, Ellingjord-Dale M, Hofvind S, Wu AH, Ursin G (2012). Physical activity and mammographic density in a cohort of postmenopausal Norwegian women; a cross-sectional study. Springer Plus..

[12] Oestreicher N, Capra A, Bromberger J, Butler LM, Crandall CJ, Gold EB (2008). Physical activity and mammographic density in a cohort of midlife women. Med Sci Sports Exerc..

[13] Peters TM, Ekelund U, Leitzmann M, Easton D, Warren R, Luben R (2008). Physical activity and mammographic breast density in the EPIC-Norfolk cohort study. Am J Epidemiol..

[14] Reeves KW, Gierach GL, Modugno F (2007). Recreational physical activity and mammographic breast density characteristics. Cancer Epidemiol Biomarkers Prev..

[15] Siozon CC, Ma H, Hilsen M, Bernstein L, Ursin G (2006). The association between recreational physical activity and mammographic density. Int J Cancer..

[16] Suijkerbuijk KP, Van Duijnhoven FJ, Van Gils CH, Van Noord PA, Peeters PH, Friedenreich CM (2006). Physical activity in relation to mammographic density in the dutch prospect-European prospective investigation into cancer and nutrition cohort. Cancer Epidemiol Biomarkers Prev..

[17] Woolcott CG, Courneya KS, Boyd NF, Yaffe MJ, Terry T, McTiernan A (2010). Mammographic density change with 1 year of aerobic exercise among postmenopausal women: a randomized controlled trial. Cancer Epidemiol Biomarkers Prev..

[18] Yaghjyan L, Colditz GA, Wolin K (2012). Physical activity and mammographic breast density: a systematic review. Breast Cancer Res Treat..

[19] Vachon CM, Kuni CC, Anderson K, Anderson VE, Sellers TA (2000). Association of mammographically defined percent breast density with epidemiologic risk factors for breast cancer (United States). Cancer Causes Control..

[20] Tyrer J, Duffy SW, Cuzick J (2004). A breast cancer prediction model incorporating familial and personal risk factors. Stat Med..

[21] KARMA (Karolinska Mammography Project for Risk Prediction of Breast Cancer). Stockholm, Sweden: Karolinska Institutet. 2011. Available from: http://karmastudy.org/sources/. Accessed 30 Nov 2014.

[22] Brand JS, Czene K, Shepherd JA, Leifland K, Heddson B, Sundbom A, et al. Automated measurement of volumetric mammographic density: a tool for widespread breast cancer risk assessment. In: Cancer epidemiology, biomarkers & prevention: a publication of the American Association for Cancer Research, cosponsored by the American Society of Preventive Oncology; 2014. vol. 23. p. 1764-72. 10.1158/1055-9965.EPI-13-1219 25012995

[23] Highnam R, Brady M, Yaffe MJ, Karssemeijer N, Harvey J (2010). Robust breast composition measurement – Volpara (TM). Lect Notes Comput Sci..

[24] Byng JW, Boyd NF, Fishell E, Jong RA, Yaffe MJ (1994). The quantitative-analysis of mammographic densities. Phys Med Biol..

[25] Bonn SE, Lagerros YT, Christensen SE, Moller E, Wright A, Sjolander A (2012). Active-Q: validation of the web-based physical activity questionnaire using doubly labeled water. J Med Internet Res..

[26] Ainsworth BE, Haskell WL, Herrmann SD, Meckes N, Bassett DR, Tudor-Locke C (2011). 2011 Compendium of physical activities: A second update of codes and MET values. Med Sci Sport Exer..

[27] Kushi LH, Doyle C, McCullough M, Rock CL, Demark-Wahnefried W, Bandera EV (2012). American Cancer Society guidelines on nutrition and physical activity for cancer prevention: reducing the risk of cancer with healthy food choices and physical activity. CA Cancer J Clin..

[28] Newey WK, McFadden D. Chapter 36 Large sample estimation and hypothesis testing. In: Robert FE, Daniel LM, editors. Handbook of econometrics. North-Holland: Elsevier; 1994. vol. 4. p. 2111-245.

[29] Evans DG, Graham J, O'Connell S, Arnold S, Fitzsimmons D (2013). Familial breast cancer: summary of updated NICE guidance. BMJ..

[30] Brand JS, Czene K, Eriksson L, Trinh T, Bhoo-Pathy N, Hall P (2013). Influence of lifestyle factors on mammographic density in postmenopausal women. PLoS One..

[31] Janiszewski PM, Ross R (2007). Physical activity in the treatment of obesity: beyond body weight reduction. Appl Physiol Nutr Metab..

[32] Miller WC, Koceja DM, Hamilton EJ (1997). A meta-analysis of the past 25 years of weight loss research using diet, exercise or diet plus exercise intervention. Int J Obes Relat Metab Disord..

[33] Stone J, Ding J, Warren RML, Duffy SW, Hopper JL (2010). Using mammographic density to predict breast cancer risk: dense area or percentage dense area. Breast Cancer Res..

[34] Vachon CM, Brandt KR, Ghosh K, Scott CG, Maloney SD, Carston MJ (2007). Mammographic breast density as a general marker of breast cancer risk. Cancer Epidemiol Biomarkers Prev..

[35] Orsini N, Bellocco R, Bottai M, Pagano M, Wolk A (2006). Age and temporal trends of total physical activity among Swedish women. Med Sci Sports Exerc..

[36] Shepherd JA, Kerlikowske K, Ma L, Duewer F, Fan B, Wang J (2011). Volume of mammographic density and risk of breast cancer. Cancer Epidemiol Biomarkers Prev..

[37] Clemons M, Goss P (2001). Estrogen and the risk of breast cancer. N Engl J Med..

[38] Friedenreich CM, Cust AE (2008). Physical activity and breast cancer risk: impact of timing, type and dose of activity and population subgroup effects. Br J Sports Med..

[39] Stolzenberg-Solomon RZ, Falk RT, Stanczyk F, Hoover RN, Appel LJ, Ard JD (2012). Sex hormone changes during weight loss and maintenance in overweight and obese postmenopausal African-American and non-African-American women. Breast Cancer Res..

[40] McTiernan A, Tworoger SS, Ulrich CM, Yasui Y, Irwin ML, Rajan KB (2004). Effect of exercise on serum estrogens in postmenopausal women: a 12-month randomized clinical trial. Cancer Res..

[41] Cauley JA, Gutai JP, Kuller LH, LeDonne D, Powell JG (1989). The epidemiology of serum sex hormones in postmenopausal women. Am J Epidemiol..

[42] Walker K, Fletcher O, Johnson N, Coupland B, McCormack VA, Folkerd E (2009). Premenopausal mammographic density in relation to cyclic variations in endogenous sex hormone levels, prolactin, and insulin-like growth factors. Cancer Res..

[43] Greendale GA, Palla SL, Ursin G, Laughlin GA, Crandall C, Pike MC (2005). The association of endogenous sex steroids and sex steroid binding proteins with mammographic density: results from the postmenopausal estrogen/progestin interventions mammographic density study. Am J Epidemiol..

[44] Bremnes Y, Ursin G, Bjurstam N, Rinaldi S, Kaaks R, Gram IT (2007). Endogenous sex hormones, prolactin and mammographic density in postmenopausal Norwegian women. Int J Cancer..

[45] Pike MC, Krailo MD, Henderson BE, Casagrande JT, Hoel DG (1983). ‘Hormonal’ risk factors, ‘breast tissue age’ and the age-incidence of breast cancer. Nature..

[46] Kelsey JL, Gammon MD, John EM (1993). Reproductive factors and breast cancer. Epidemiol Rev..

[47] Eliassen AH, Missmer SA, Tworoger SS, Spiegelman D, Barbieri RL, Dowsett M (2006). Endogenous steroid hormone concentrations and risk of breast cancer among premenopausal women. J Natl Cancer Inst..

[48] Tworoger SS, Missmer SA, Barbieri RL, Willett WC, Colditz GA, Hankinson SE (2005). Plasma sex hormone concentrations and subsequent risk of breast cancer among women using postmenopausal hormones. J Natl Cancer Inst..

[49] Bernstein L, Ross RK, Lobo RA, Hanisch R, Krailo MD, Henderson BE (1987). The effects of moderate physical-activity on menstrual-cycle patterns in adolescence – implications for breast-cancer prevention. Br J Cancer..

[50] Sternfeld B, Jacobs MK, Quesenberry CP, Gold EB, Sowers M (2002). Physical activity and menstrual cycle characteristics in two prospective cohorts. Am J Epidemiol..

[51] Tworoger SS, Missmer SA, Eliassen AH, Barbieri RL, Dowsett M, Hankinson SE (2007). Physical activity and inactivity in relation to sex hormone, prolactin, and insulin-like growth factor concentrations in premenopausal women – exercise and premenopausal hormones. Cancer Causes Control..

[52] McTiernan A, Wu L, Chen C, Chlebowski R, Mossavar-Rahmani Y, Modugno F (2006). Relation of BMI and physical activity to sex hormones in postmenopausal women. Obesity..

[53] Warren MP, Perlroth NE (2001). The effects of intense exercise on the female reproductive system. J Endocrinol..

[54] Bray F, McCarron P, Parkin DM (2004). The changing global patterns of female breast cancer incidence and mortality. Breast Cancer Res..

[55] Nordic Counsil of Ministers. Nordic Nutrition Recommendations 2012 – integrating nutrition and physical activity. 2014. http://dx.doi.org/10.6027/Nord2014-002. Accessed 30 Nov 2014.

